# Personal agency and borderline personality disorder: a longitudinal study of outcomes

**DOI:** 10.1186/s12888-022-04214-5

**Published:** 2022-08-22

**Authors:** Talia Hashworth, Samantha Reis, Michelle Townsend, Jessica O.’Garr, Brin F.S. Grenyer

**Affiliations:** 1 School of Psychology, University of Wollongong, Wollongong, Australia; 2 Illawarra Health and Medical Research Institute, University of Wollongong, Wollongong , Australia; 3 South Coast Private Mental Health Hospital, Wollongong, Australia

**Keywords:** Personal agency, Locus of control, Borderline personality disorder, Dialectical behaviour therapy, Group therapy, Treatment outcomes

## Abstract

**Background:**

Low personal agency is the concept of attributing successes and failures to external factors rather than personal characteristics. Previous research supported links between low personal agency and symptoms of borderline personality disorder (BPD). The present research followed patients in an outpatient dialectical behavioural therapy (DBT) group from intake to 12 months follow up to examine the impact of personal agency on outcome.

**Methods:**

Patients (*N* = 57, age 18–72, 91.5% female) were assessed at intake, after three months of DBT treatment, and 12 months follow up on measures of symptoms and personal agency. Three separate measures were used to assess treatment outcomes: the BPD Checklist, the Personality Inventory for DSM-5 (PID-5), and the Mental Health Inventory (MHI-5).

**Results:**

Mixed model analyses found BPD symptoms significantly reduced as a result of DBT treatment and were maintained at follow-up. However, 47% of participants continued to meet BPD criteria 12 months later, despite treatment. Regression analyses indicated that low personal agency at intake was associated with higher BPD symptom severity at post-treatment and 12 month follow up. In addition, low personal agency at intake was associated with greater levels of negative affectivity at post-treatment. Personal agency did not relate to levels of depression and anxiety.

**Conclusions:**

Despite the reductions in BPD symptomology, personal agency did not significantly change over time. Those with lower agency at intake continued to do more poorly at follow up. We speculate that poor outcomes may be contributed to by patients' lack of engagement in recovery due to poor agency and an external locus of control. As such, therapeutic approaches, like DBT, may require additional strategies to appropriately target low personal agency. Further research is needed to understand if other treatment protocols may facilitate positive change in personal agency.

## Background

Borderline personality disorder (BPD) is a psychological disorder characterised by impairments of self and interpersonal functioning [1]. Individuals with BPD tend to experience and display pervasive patterns of interpersonal instability [1]. Particularly, individuals with BPD may experience fears of abandonment, intense interpersonal relationships, identity disturbances, impulsivity, self-harm or suicidal behaviours, emotional lability, emptiness, difficulty controlling anger, and dissociation [1]. Previous research highlighted that depression and anxiety were highly comorbid with the presentation of BPD [2–4]. Previous research found that individuals with BPD demonstrate greater severity of depression and anxiety symptoms compared to those with other personality disorders and those with a mood disorder only [4], whereas others [2, 3] found no significant differences in depression severity for those with comorbid BPD and depression.

The evidence base for treating BPD has, over time, demonstrated modest reductions in symptoms following treatment BPD [5–7]. Dialectical behavioural therapy (DBT) is considered a good psychotherapeutic treatment for BPD targeting four key areas – mindfulness, interpersonal effectiveness, distress tolerance, and emotion regulation [6]. A review examining the efficacy of psychotherapy for BPD found that DBT and psychodynamic approaches were moderately more effective than control interventions (e.g. CBT, supportive therapy) [7]. Authors further note high levels of publication bias and significant differences in duration and type of follow-up design. In addition, meta-analytic reviews note there are some reported benefits in reduced suicidality and self-harm, increased emotion regulation and problem-solving abilities [8]. Studies of 75 RCTs have shown DBT and mentalization-based treatment (MBT) were the primary interventions studied and results found significant differences in the reduction of BPD symptoms using both of these approaches compared to treatment as usual (TAU) [9]. It was further explained that these therapeutic approaches may reduce depression symptoms compared to TAU but this finding was uncertain [9].

### Personal agency

Personal agency represents the type of effortful behaviour a person makes towards maintaining and improving their circumstances and may relate to the experience of BPD and the degree to which people engage in treatment and recovery from mental health challenges. It can be operationalized on a continuum, where high personal agency relates to an internal locus of control (LOC) focus, and low personal agency corresponds with external LOC focus. Locus of control (LOC) refers to the degree that individuals attribute successes and failures to themselves or others [10]. Research has showed that individuals presenting with high personal agency tended to attribute outcomes as dependent on their own personal characteristics (e.g., the effort that they put into a task), while those who presented with low personal agency often believed outcomes were a direct result of external factors (e.g., luck, chance, or fate) [11, 12]. There has been conflicting evidence on the stability of personal agency over time. Some studies [13–15] suggested that personal agency is a trait that is stable over life, while other studies [16, 17] proposes that this may change over time.

Previous research suggested that individuals with low levels of personal agency tend to experience greater psychopathology [18–23]. Scant research expands this knowledge into the relationship between personal agency and BPD specifically [24–28]. A recent study found that greater personal agency was associated with lower levels of BPD symptoms [29]. Two studies examined changes in personal agency following therapeutic intervention in clinical samples [30, 31]. Both studies found that following treatment, individuals reported significant changes in personal agency and greater control over their emotions. In a community sample, lower personal agency was associated with greater BPD symptomology, as well as with insecure adult attachment styles [24]. An association was also found between low personal agency and high BPD symptomology in studies utilising community samples [26], while Watson [25] found an association between low personal agency and personality disorders symptoms in general.

The relationships between personal agency and broader symptoms beyond BPD such as pathological personality traits and mood and anxiety symptoms have been less explored. An older study suggested that greater self-regulation (which is related to higher personal agency) can mediate the effects of negative affect [32]. Understanding the impact of personal agency on pathological personality traits and mood and anxiety may be relevant as previous studies indicate that both of these factors may negatively impact treatment outcome [33–37]. Despite the previous literature examining BPD and personal agency [24, 26, 28, 29, 31], little is known about how these two constructs interact. In addition, there is minimal knowledge regarding the influence of personal agency on treatment outcomes within a clinical sample. The present study addressed these gaps in the literature by examining personal agency within individuals with BPD undergoing a DBT program and assessing the role of personal agency in treatment outcomes. First, it was expected that BPD symptom severity will significantly reduce over the course of the DBT treatment. Previous longitudinal studies have shown changes in BPD symptom severity following treatment; however, there were inconsistencies on whether these changes remain over time [29, 38]. Secondly, consistent with previous research [24–28], it was expected that there will be a relationship between personal agency and BPD, such that low personal agency will be associated with greater BPD symptomology. Finally, given research suggesting that personal agency may be relevant to the experience of BPD, it was hypothesized greater personal agency may be associated with more marked changes in BPD symptomology after therapy and at 12-month follow-up. No previous studies have examined the impact of personal agency on BPD symptoms over time and in response to therapy.

## Method

### Aim and study design

The present study was structured as a pilot study and aimed to address gaps in the literature by examining personal agency within individuals with BPD undergoing a DBT program and assessed the role of personal agency in treatment outcomes.

### Treatment

Patients with Borderline Personality Disorder attended a three-month outpatient DBT group at a local hospital where they gave written informed consent to participate. The psychiatrist conducted an initial assessment with the patient and provided a diagnosis prior to attending the DBT program. Following this, the treating psychologist and the researcher worked together to confirm or amend the diagnosis based on clinical symptoms present during the program along with results of self-report questionnaires. From this, the psychiatrist, psychologist, and researcher together finalised a diagnosis. The group was semi-open, where new participants entered every four weeks during a mindfulness session. Standard DBT protocols were administered in the outpatient program [6, 39]. The program was run by a clinical psychologist and an intern psychologist once a week for five and a half hours. The breakdown of the program was as follows: 1) morning session for an hour and a half for homework review, 2) 30-min break, 3) middle session for an hour and a half for didactic learning and skills training, 4) 30-min lunch break, 5) afternoon session for an hour and a half for skills training and homework setting. Mindfulness skills were used in each session. Manuals were followed for adherence to validated DBT protocols, and two DBT trained authors lead the groups. In addition, therapeutic alliance checks were independently made to ensure treatment was well received by patients. Therapeutic alliance was assessed using a brief form of the Penn Helping Alliance Questionnaire [40]. At post-treatment, results showed that 93% of participants felt their therapists were warm, supportive, and helpful and 92% of participants felt as though they worked well with their therapists.

### Participants

The sample consisted of 57 adults (Mean age = 35.86 years, SD = 13.61, Range = 18–72). Patients all had current or lifetime BPD and various comorbid diagnoses. Comorbid diagnoses included depression, anxiety, PTSD, and bipolar disorder, diagnosed by the treating psychologist and psychiatrist. The sample was predominately female (91.5% at intake, 92.1% at post-treatment, and 90.5% at follow-up). The study had an attrition rate with 23.37% of the sample withdrawing from intake to follow-up. Post treatment assessment retained 37 adults (mean age = 34.81, SD = 11.65, range = 19–56), and 41 adults (mean age = 37.80, SD = 13.32, range = 20–72) were assessed at 12-month follow-up. Four were unable to be assessed at post treatment were able to be assessed at 12 months, leading to a slightly higher follow-up rate. Those who dropped out of the study or were unable to be assessed did not significantly differ when compared on intake clinical characteristics to those who were retained according to independent samples t-tests on measures of personal agency (*p* = 0.57), BPD (*p* = 0.20), and MHI-5 (*p* = 0.95) or on demographic details such as gender (*p* = 0.06) and age (*p* = 11.13). Further demographic details are indicated in Table 1.

**Table 1:** Sample characteristics at baseline for full sample (*N* = 57)

Demographic	Item	N	Percentage
Age		57	M = 35.86, SD = 13.61
Gender	Female	53	93.00
	Male	4	7.00
In a relationship	No (single)	26	45.60
	Yes	31	54.40
Highest level of education	High school	14	24.60
	Trade Certificate	17	29.80
	College Degree	26	45.70
Who do you live with?	Alone	7	12.30
	Alone with child(ren)	5	8.80
	Friends	5	8.80
	Parents	12	21.10
	Spouse/partner	14	24.60
	Spouse/partner and child(ren)	8	14.00
	Other relatives	6	10.60
Do you have children?	No	33	57.90
	Yes	24	42.10

#### Depression and anxiety

The Mental Health Inventory (MHI-5) [41] was used to assess current depression and anxiety symptoms. The MHI-5 was a five-item measure and is one of the domains of the Short Form 36 (SF-36) [42]. It asked how participants have been feeling over the past two weeks. Examples of questions include, “Have you been a very nervous person?” Each item had six alternative answers, ranging from “all of the time” to “not at all”, with a sum score from 5 to 30. The scores were then transformed to a 0–100 scale with high scores indicating good mental health [41]. Previous researchers [43] reported the internal consistency in their community sample to be high at α = 0.84 in a community sample. In the current study, the MHI-5 was used as a continuous variable and reliability analyses were α = 0.74 for intake, α = 0.82 for post-treatment, and α = 0.83 for follow-up.

#### Personal agency

The Mental Health Locus of Control Scale (MH-LOC) [44] measured personal agency. Scores (22–132) can be understood as indicating internal locus of control or external locus of control, with scores of 77 at the midpoint. Low scores reflected a more internal locus of control (greater personal agency) and high scores reflected a more external locus of control (poorer personal agency). In the original study (44), the mean scores of participants was 74.14 (SD = 11.19), α = 0.84. Reliability analyses of the MH LOC for the current full sample were α = 0.56 for intake, α = 0.76 for post-treatment, and α = 0.70 for follow-up.

#### BPD symptoms

Presence and severity of BPD symptoms were assessed using the Borderline Personality Disorder Checklist (BPD CL) [60]. A cut-off score of 7 or higher on the MSI-BPD indicated a likely diagnosis of BPD and suggested high sensitivity (0.81) and specificity (0.85). Reliability analyses for the full sample for study three were α = 0.96 for intake, α = 0.97 for post-treatment, and α = 0.94 for follow-up.

#### Pathological personality traits

The Personality Inventory Brief Form for the DSM-5 (PID-5-BF) [45] was used. It comprises 25 items that assess maladaptive personality traits. Items were rated based on how accurately they describe participants from 0 (very false or often very false) to 3 (very true or often true). Each subscale consists of five items and are: negative affectivity (e.g., “I worry about almost everything”), detachment (e.g., “I don’t like to get too close to people”), antagonism (e.g., “It’s no big deal if I hurt other peoples’ feelings”), disinhibition (e.g., “I feel like I act totally on impulse”), and psychoticism (e.g., “I have seen things that weren’t really there”) [45]. Higher total scores were indicative of greater overall personality dysfunction, ranging from 0 to 75 [45]. In addition, each subscale ranged in scores from 0 to 15, with higher scores indicating greater dysfunction in the specific personality trait domain [45]. All subscales demonstrated high reliability. At intake, reliability estimates were: negative affectivity α = 0.77, detachment at α = 0.62, antagonism at α = 0.78, disinhibition at α = 0.81, and psychoticism at α = 0.9. At post-treatment, reliability for negative affectivity was recorded as α = 0.86, detachment at α = 0.76, antagonism at α = 0.52, disinhibition at α = 0.82, and psychoticism at α = 0.86.

#### Data analysis

Data was collected at three time points – intake, post-treatment and at follow-up. Intake data was collected prior to participants first treatment session, post-treatment collected 12 weeks after intake, and follow-up collected 12 months after intake. Data were de-identified and inputted into SPSS statistical analysis software. Patients were grouped into two classifications of currently meeting BPD criteria (scores of 100 or greater) or not currently meeting BPD criteria (scores of 99 or lower) using the BPD CL. Mixed model analyses were performed including all data across the time points, with BPD symptoms as the dependent variable. Linear regression analyses were used to examine the role of personal agency on treatment outcome measures. A Bonferroni correction of the p-value was used to correct for multiple testing. For each correlation, seven possible tests were conducted (BPD symptoms, MHI-5 scores, and PID-5 domains: negative affect, detachment, disinhibition, antagonism, psychoticism. The adjusted *p*-value was 0.007 with the α of 0.05 and the level of comparisons as 7. Using this adjusted *p*-value, personal agency at intake is still correlated with BPD symptoms. In addition, personal agency at intake did not correlate with any post-treatment or follow-up measures using the adjusted *p*-value.

## Results

A mixed model analysis was used to determine changes in BPD symptoms over time. Data were reformatted based on three time points with BPD symptoms as the dependent variable and time as the factor [46]. Results found significant reductions in BPD scores over the course of the DBT group program. Results suggest a significant reduction of BPD scores on the BPD Checklist by 17.04 points from intake to post-treatment (b = -17.04, t = -2.25, *p* = 0.03, 95%CI[-32.02, -2.05]). Results remained stable at follow-up, with a reduction of 17.21 points from intake to follow-up (b = -17.21, t = -2.37, *p* = 0.02, 95%CI[-31.60,-2.82]). Results suggest that BPD symptom severity significantly decreased as a result of treatment and remained stable over 12 months.

In addition, the distribution of participants meeting BPD criteria over time was explored using the full sample. Results demonstrated across the entire sample, at intake, approximately 65% of participants met criteria for BPD based on scores of 100 or greater on the BPD CL. At post-treatment, 40% of the sample meet criteria for BPD and at follow-up, 47.5% meet criteria for BPD. When looking at only the participants who met criteria for BPD at intake, approximately 71% still met criteria for BPD at post-treatment and 78% still met criteria for BPD at follow-up. These results showed that over time, the number of participants meeting criteria for BPD reduced, with less than half of the sample still meeting criteria at follow-up. In addition, the majority of participants who met criteria at intake continued to meet criteria over time, supporting previous research that has demonstrated challenges in achieving remission over time [47].

To assess whether personal agency influenced treatment outcomes, specifically, BPD symptoms, a hierarchical regression analysis was used to examine the role of personal agency on BPD symptom severity over time. The first block was BPD scores at intake, as this variable was controlled for and the second block was personal agency at intake. Personal agency at intake was used as the independent variable and BPD scores at post-treatment was the dependent variable. The results of the first block revealed that BPD scores at intake was significant and explained 49% of the variance [(R^2^ = 0.49, F(1, 33) = 31.88, *p* = 0.00)]. The second block also revealed a significant model and explained 50% of the variance [(R^2^ = 0.50, F(2, 32) = 15.98, *p* = 0.00)]. These results suggest that when controlling for intake BPD severity, personal agency at intake was associated with BPD scores at post-treatment.

Next, a hierarchical regression analysis was also used to examine the role of personal agency on BPD symptom severity at follow-up. The first block was BPD scores at intake, as this variable was controlled for and the second block was personal agency at intake. Personal agency at intake was used as the independent variable and BPD scores follow-up was the dependent variable. The results of the first block revealed that BPD scores at intake was significant and explained 30% of the variance [(R^2^ = 0.30, F(1, 38) = 16.04, *p* = 0.00)]. The second block also revealed a significant model and explained 32% of the variance [(R^2^ = 0.32, F(2, 37) = 8.65, *p* = 0.00)]. These results suggest that when controlling for intake BPD severity, personal agency at intake may also be associated with BPD scores at 12 month follow-up.

Pathological personality traits and levels of depression and anxiety were also used as treatment outcomes. A linear regression was used to examine the role of personal agency on anxiety and depression symptom severity over time. All assumptions specific to each statistical test were checked prior to analyses. All variables displayed violations of normality. No outliers were detected and tests for assumptions of linearity, multicollinearity, and homogeneity of variance were satisfied. Personal agency at intake was used as the independent variable and the MHI-5 scores at post-treatment was the dependent variable. Results indicated that personal agency at intake was not associated with depression and anxiety symptom severity at post-treatment [(R^2^ = 0.03, F(1, 25) = 0.82, *p* = 0.38)]. Next, a linear regression analysis examined personal agency at intake with depression and anxiety symptoms at follow-up. Results similarly found that personal agency at intake was not associated with BPD symptom severity after 12 months [(R^2^ = .-0.02, F(1, 38) = 0.41, *p* = 0.53)]. These regression analyses suggest that personal agency at intake was not associated with levels of depression and anxiety.

Furthermore, a linear regression was also used to examine the role of personal agency on the severity of pathological personality traits over time. Personal agency at intake was used as the independent variable and the PID-5 total scores at post-treatment was the dependent variable. Results indicated that personal agency at intake was not associated with the severity of pathological personality traits at post-treatment [(R^2^ = -0.01, F(1, 34) = 0.64, *p* = 0.43)]. Next, further regression analyses were used to examine the role of personal agency on the specific domains of personality pathology at post-treatment. Results indicated personal agency at intake was associated with negative affectivity at post-treatment [R^2^ = 0.12, F(1, 34) = 5.96, *p* = 0.02)]. Personality pathology was not assessed at follow-up and thus no associations can be made.

To understand the cross-sectional relationship between intake personal agency and other measures, correlational analyses were undertaken. Personal agency at intake correlated with the following intake measures: BPD symptoms (*r* = 0.32, *p* = 0.01) and negative affect (*r* = 0.26, *p* = 0.05). Personal agency at intake did not relate to BPD symptoms (*r* = 0.18, *p* = 0.31) or the MHI-5 (*p* = 0.38) at post-treatment. Personal agency at intake correlated with negative affect (*r* = 0.39, *p* = 0.02) at post-treatment. Personal agency at intake correlated with BPD symptoms (*r* = 0.33, *p* = 0.04) at follow-up but did not correlate with the MHI-5 (*r* = 0.10, *p* = 0.53). The PID-5 was not assessed at follow-up.

### Does personal agency change during treatment?

Personal agency did not change over time, with mean scores at intake of 80.19, post treatment 78.19, and follow up 81.93 (Table 2). Statistical analysis showed no change from intake to post-treatment (M = -0.17, SD = 11.64, t = -0.09, df = 35, *p* = 0.93) or intake to follow-up (M = 2.35, SD = 13.62, t = 1.09, df = 39, *p* = 0.28) (Table 2). Overall, no significant shifts occurred in personal agency, despite changes in BPD severity, suggesting in this sample it was stable and resistant to change.

**Table 2:** Clinical outcomes for intake, post-treatment, and follow-up

	Intake (*N* = 57)		Post-Treatment (*N* = 36)		Follow- Up (*N* = 40)		Intake to Post-Treatment		Intake to Followup	
	M	SD	M	SD	M	SD	d	p	d	p
BPD CL	117.81	36.74	100.77	36.54	100.6	31.79	.47	.00*	.50	.03*
MH LOC	80.19	9.78	78.19	12.40	81.93	13.34	.18	.93	.15	.28
MHI-5	64.49	20.14	49.56	18.27	50.6	20.9	.78	.00*	.68	.00*
PID-5	32.04	12.38	26.08	13.02			.47	.00*		
Negative affect	9.69	3.62	8.19	4.21			.38	.00*		
Detachment	7.15	3.36	5.81	3.45			.39	.01*		
Antagonism	2.53	2.41	1.89	1.85			.30	.04*		
Disinhibition	5.90	4.04	4.94	3.69			.25	.02*		
Psychoticism	6.79	4.08	5.83	4.35			.23	.10*		

*BPD CL* Borderline Personality Disorder Checklist, *MH LOC* Mental Health Locus of Control Scale

*MHI-5* The Mental Health Inventory, *PID-5* The Personality Inventory Brief Form for the DSM-5

^*^ = *p* < .05

## Discussion

This pilot study followed patients attending group DBT from intake through three-month termination and 12 month follow up. As expected, DBT therapy led to significant reductions in BPD symptoms over time, which were maintained at follow up. In addition, depression and anxiety symptoms did not significantly reduce as a result of treatment. Given these results, it is possible that this form of outpatient DBT group may be successful in reducing BPD symptoms but may not be as effective in treating depression and anxiety symptoms. The lack of significant change in mood disorder symptoms from this study were contradictory to previous research [48, 49]. This may be explained by the large focus on BPD symptoms in group settings, as individual mood and anxiety symptoms would not be explored and targeted.

When controlling for BPD scores at intake, personal agency showed a significant relationship to BPD symptom scores at post-treatment and follow-up. Personal agency at intake was not however associated with depression and anxiety symptoms at any of the three time points. Personal agency at intake was also associated with higher levels of PID-5 negative affectivity at post-treatment. These results highlight the important role of personal agency, not only at the start of treatment, but also throughout the intervention, and suggest this may be a key factor influencing therapeutic change. These results were consistent with previous research, which indicated that low personal agency was associated with greater BPD symptom severity [29]. In addition, these results were consistent with studies indicating high personal agency is associated with successful treatment outcomes [50–52]. It is important to note that these correlational results may also be due to an underlying third variable, for example, adverse childhood experiences, schemas, or comorbid diagnoses. As a result, the correlational analyses should be interpreted with caution.

Almost half of the sample still met criteria for BPD at follow-up (47%). In addition, the majority of participants who met criteria at intake continued to meet criteria over time. A recent systematic review found that approximately half of the sample did not respond to treatment for BPD, which may be consistent with the results of this study [47]. It was unclear if the participants in the study did not respond to DBT protocols or if they did respond but were not yet recovered. As discussed, the definition of recovery in BPD was unclear and needs further exploration [53–57]. In this study, it is possible that DBT may have be reducing symptoms of BPD but three months of treatment may not be effective enough to obtain full recovery from the diagnosis. In addition, since personal agency has been identified as one of the key factors that supports recovery from BPD [54], further research may be needed to understand the role of personal agency in BPD recovery if personal agency is resistant to change.

### Strengths and limitations

There are a number of limitations worth noting. First, due to hospital requirements, it was not possible to conduct independent clinical or diagnostic interviews on patients prior to their participation in the study. To account for this limitation, the diagnostic presentations and suitability were verified with the treating psychologist and referring psychiatrist, as described in the Method section. Second, as indicated in previous research [11, 24], the MH LOC has limited research around its use, particularly in clinical populations. Despite this, it was reliable and the face validity was acceptable. Third, it was unclear whether participants sought additional medical or psychological support over the 12 months following treatment. It is possible that results at follow-up may be influenced by further care. Fourth, the small sample size may impact the generalizability of the results of this study. Fifth, the nature of the DBT protocols administered may not be comparable in an inpatient and outpatient setting. Additionally, BPD symptoms may present as more severe in an inpatient setting along with lower psychosocial functioning. As a result, it is possible that the results of this study may not be generalizable to an inpatient sample of individuals with BPD. Finally, though this sample consisted of clinical treatment-seeking individuals, a control group was not utilised. As a result, findings should be interpreted with caution and may not be generalisable to other samples and other treatments.

### Clinical implications and future research

The novelty of these findings can assist in filling a gap in the literature and may benefit clinicians treating people with BPD. We suggest that clinicians may benefit from assessing personal agency at different points throughout therapy while also making conscious efforts to increase their patient’s sense of autonomy, self-responsibility, and self-control. Regular assessment will further determine whether personal agency is stable over time and this needs additional study. Particular clinical strategies that may be useful in identifying and increasing personal agency include work that suggests increasing mindful awareness of strengths and limitations, and cognitive restructuring can be helpful [58, 59]. From the results of the study, clinicians may find that understanding personal agency levels in the early stages of treatment may assist in being able to anticipate degree of change over time. This may be an asset for clinicians to be able to approximate the duration or frequency of treatment needed, according to levels of personal agency.

It may be beneficial for future research to examine whether ongoing treatment over time would impact personal agency and BPD symptoms compared to those who do not continue longer term treatment. In addition, future research may consider developing a treatment protocol for individuals with BPD that specifically targets levels of personal agency. To conclude, low personal agency can be understood to be related to greater BPD but may not be related to mood and anxiety symptoms. In addition, personal agency may be a stable variable and may be resistant to change. The results of this study suggest that personal agency may be associated with BPD symptom severity over time, which may clinically inform the duration and frequency of treatment for individuals with BPD. The authors highlight the preliminary nature of these results and recommend further replication. Future research is needed to further understand the link between low personal agency and BPD.

## Conclusions

Low personal agency at the start of treatment appeared to inhibit treatment progress over 12 months. Within this study, personal agency was stable and did not significantly change as a result of treatment. This suggests that personal agency may be resistant to change following DBT treatment. It may be beneficial for future research to develop a treatment protocol that may be able to target and increase personal agency more specifically. Though DBT has been shown to be effective in treating BPD [7–9], no known research illustrates whether it may be effective in improving personal agency. In the present study, participants level of personal agency did not significantly increase or decrease as a result of treatment.

## Data Availability

The data is not available as participants only gave ethical consent for this project, and not for further distribution outside the research team. The authors are under ethical obligations not to release the data to third parties. Access to the data would require the approval of the joint Human Research Ethics Committee of the University of Wollongong and NSW Health. Applications to access the data may be sent to Ethics Manager, 1 Northfields Avenue, University of Wollongong NSW 2522 Australia, rso@uow.edu.au.

## Abbreviations

BPD: Borderline Personality Disorder
LOC: Locus of Control
DBT: Dialectical Behaviour Therapy
TAU: Treatment as Usual
MBT: Mentalization-based Treatment
PTSD: Posttraumatic Stress Disorder
BPD CL: Borderline Personality Disorder Checklist
MH LOC: The Mental Health Locus of Control Scale
MHI-5: The Mental Health Inventory
PID-5: The Personality Inventory Brief Form for the DSM-5

## References

[1] APA (2013). Diagnostic and statistical manual of mental disorders (DSM-5®): American Psychiatric Pub.

[2] Stanley B, Wilson ST (2006). Heightened subjective experience of depression in borderline personality disorder. J Pers Disord.

[3] Köhling J, Ehrenthal JC, Levy KN, Schauenburg H, Dinger U (2015). Quality and severity of depression in borderline personality disorder: a systematic review and meta-analysis. Clin Psychol Rev.

[4] Comtois KA, Cowley DS, Roy-Byrne PP (1999). Relationship between borderline personality disorder and Axis I diagnosis in severity of depression and anxiety. J Clin psychiatry.

[5] Grenyer BF (2013). Improved prognosis for borderline personality disorder. Med J Aust.

[6] Linehan M (2014). DBT Skills training manual: Guilford Publications.

[7] Cristea IA, Gentili C, Cotet CD, Palomba D, Barbui C, Cuijpers P (2017). Efficacy of psychotherapies for borderline personality disorder: a systematic review and meta-analysis. JAMA Psychiat.

[8] Stoffers-Winterling JM, Storebø OJ, Kongerslev MT, Faltinsen E, Todorovac A, Jørgensen MS (2022). Psychotherapies for borderline personality disorder: a focused systematic review and meta-analysis. Br J Psychiatry.

[9] Storebø OJ, Stoffers-Winterling JM, Völlm BA, Kongerslev MT, Mattivi JT, Jørgensen MS (2020). Psychological therapies for people with borderline personality disorder. Cochrane Database Syst Rev.

[10] Rotter JB, Chance JE, Phares EJ (1972). Applications of a social learning theory of personality.

[11] Lefcourt HM (1991). Locus of control: Academic Press.

[12] Lekfuangfu WN, Powdthavee N, Warrinnier N, Cornaglia F (2018). Locus of control and its intergenerational implications for early childhood skill formation. Econ J.

[13] Kulas H (1996). Locus of control in adolescence: a longitudinal study. Adolescence.

[14] Anderson CR (1977). Locus of control, coping behaviors, and performance in a stress setting: a longitudinal study. J Appl Psychol.

[15] Craighead WE, Nemeroff CB (2000). The Corsini Encyclopedia of Psychology and Behavioral Science, Volume 4: Wiley.

[16] Cobb-Clark DA, Schurer S (2013). Two economists’ musings on the stability of locus of control. Econ J.

[17] Legerski EM, Cornwall M, O'Neil B (2006). Changing locus of control: Steelworkers adjusting to forced unemployment. Soc Forces.

[18] Coyne LW, Thompson AD (2011). Maternal depression, locus of control, and emotion regulatory strategy as predictors of preschoolers’ internalizing problems. J Child Fam Stud.

[19] Culpin I, Stapinski L, Miles ÖB, Araya R, Joinson C (2015). Exposure to socioeconomic adversity in early life and risk of depression at 18 years: the mediating role of locus of control. J Affect Disord.

[20] Omani Samani R, Maroufizadeh S, Navid B, Amini P (2017). Locus of control, anxiety, and depression in infertile patients. Psychol Health Med.

[21] Karimi R, Alipour F (2011). Reduce job stress in organizations: Role of locus of control. Int J Bus Soc Sci.

[22] Buddelmeyer H, Powdthavee N (2016). Can having internal locus of control insure against negative shocks? psychological evidence from panel data. J Econ Behav Organ.

[23] Stillman S, Velamuri M (2016). If life throws you lemons try to make lemonade: does locus of control help people cope with unexpected shocks. Available at SSRN 2835350.

[24] Hashworth T, Reis S, Grenyer BF (2021). personal agency in borderline personality disorder: the impact of adult attachment style. Front Psychol.

[25] Watson DC (1998). The relationship of self-esteem, locus of control, and dimensional models to personality disorders. J Soc Behav Pers.

[26] Hope NH, Wakefield MA, Northey L, Chapman AL (2018). The association between locus of control, emotion regulation and borderline personality disorder features. Personal Ment Health.

[27] Bateman AW, Gunderson J, Mulder R (2015). Treatment of personality disorder. The Lancet.

[28] Pinto A, Grapentine WL, Francis G, Picariello CM (1996). Borderline personality disorder in adolescents: Affective and cognitive features. J Am Acad Child Adolesc Psychiatry.

[29] Bernheim D, Gander M, Keller F, Becker M, Lischke A, Mentel R (2019). The role of attachment characteristics in dialectical behavior therapy for patients with borderline personality disorder. Clin Psychol Psychother.

[30] Schuppert HM, Giesen-Bloo J, van Gemert TG, Wiersema HM, Minderaa RB, Emmelkamp PM (2009). Effectiveness of an emotion regulation group training for adolescents—a randomized controlled pilot study. Clini Psychol & Psychother.

[31] Löffler-Stastka H, Ponocny-Seliger E, Meißel T, Springer-Kremser M (2006). Gender aspects in the planning of psychotherapy for borderline personality disorder. Wien Klin Wochenschr.

[32] Bandura A (1999). A sociocognitive analysis of substance abuse: an agentic perspective. Psychol Sci.

[33] Ramos-Grille I, Gomà-i-Freixanet M, Aragay N, Valero S, Vallès V (2015). Predicting treatment failure in pathological gambling: the role of personality traits. Addict Behav.

[34] Ingram SH, South SC (2021). The longitudinal impact of DSM–5 Section III specific personality disorders on relationship satisfaction. Personal Disord Theory Res Treat.

[35] Choate AM, Gorey C, Rappaport LM, Wiernik BM, Bornovalova MA (2021). Alternative model of personality disorders traits predict residential addictions treatment completion. Drug Alcohol Depend.

[36] Williams MM, Rogers R (2019). Stigma experiences of patients with problematic personality traits: an investigation with the PID-5. Stigma and Health.

[37] Fowler JC, Madan A, Allen JG, Oldham JM, Frueh BC (2019). Differentiating bipolar disorder from borderline personality disorder: diagnostic accuracy of the difficulty in emotion regulation scale and personality inventory for DSM-5. J Affect Disord.

[38] Turner RM (2000). Naturalistic evaluation of dialectical behavior therapy-oriented treatment for borderline personality disorder. Cogn Behav Pract.

[39] Linehan MM, Comtois KA, Murray AM, Brown MZ, Gallop RJ, Heard HL (2006). Two-year randomized controlled trial and follow-up of dialectical behavior therapy vs therapy by experts for suicidal behaviors and borderline personality disorder. Arch Gen Psychiatry.

[40] Luborsky L, Barber JP, Siqueland L, Johnson S, Najavits LM, Frank A (1996). The revised helping alliance questionnaire (HAq-II): psychometric properties. J Psychother Pract Res.

[41] Berwick DM, Murphy JM, Goldman PA, Ware JE, Barsky AJ, Weinstein MC (1991). Performance of a five-item mental health screening test. Medical care.

[42] Ware JE, Sherbourne CD (1992). The MOS 36-item short-form health survey (SF-36): I. conceptual framework and item selection. Med Care.

[43] McCabe CJ, Thomas KJ, Brazier JE, Coleman P (1996). Measuring the mental health status of a population: a comparison of the GHQ–12 and the SF–36 (MHI–5). Br J Psychiatry.

[44] Hill DJ, Bale RM (1980). Development of the mental health locus of control and mental health locus of origin scales. J Pers Assess.

[45] Krueger R, Derringer J, Markon K, Watson D, Skodol A (2013). The personality inventory for DSM-5—brief form (PID-5-BF)—adult.

[46] Gueorguieva R, Krystal JH (2004). Move over anova: progress in analyzing repeated-measures data andits reflection in papers published in the archives of general psychiatry. Arch Gen Psychiatry.

[47] Woodbridge J, Townsend M, Reis S, Singh S, Grenyer BF (2021). Non-response to psychotherapy for borderline personality disorder: a systematic review. Aust N Z J Psychiatry..

[48] Keuthen NJ, Rothbaum BO, Fama J, Altenburger E, Falkenstein MJ, Sprich SE (2012). DBT-enhanced cognitive-behavioral treatment for trichotillomania: a randomized controlled trial. J Behav Addict.

[49] Malivoire BL (2020). Exploring DBT skills training as a treatment avenue for generalized anxiety disorder. Clin Psychol Sci Pract.

[50] Balch P, Ross AW (1975). Predicting success in weight reduction as a function of locus of control: a unidimensional and multidimensional approach. J Consult Clin Psychol.

[51] McAnena C, Craissati J, Southgate K (2016). Exploring the role of locus of control in sex offender treatment. J Sex Aggress.

[52] Rotter JB (1966). Generalized expectancies for internal versus external control of reinforcement. Psychol Monogr Gen Appl.

[53] Katsakou C, Marougka S, Barnicot K, Savill M, White H, Lockwood K (2012). Recovery in borderline personality disorder (BPD): a qualitative study of service users' perspectives. PLoS ONE.

[54] Ng FY, Townsend ML, Miller CE, Jewell M, Grenyer BF (2019). The lived experience of recovery in borderline personality disorder: a qualitative study. Borderline personality disorder and emotion dysregulation.

[55] Zanarini MC, Frankenburg FR, Reich DB, Fitzmaurice G (2010). Time to attainment of recovery from borderline personality disorder and stability of recovery: a 10-year prospective follow-up study. Am J Psychiatry.

[56] Zanarini MC, Frankenburg FR, Reich DB, Fitzmaurice G (2012). Attainment and stability of sustained symptomatic remission and recovery among patients with borderline personality disorder and axis II comparison subjects: a 16-year prospective follow-up study. Am J Psychiatry.

[57] Soloff PH (2021). Bridging the gap between remission and recovery in BPD: qualitative versus quantitative perspectives. J Pers Disord.

[58] Hamarta E, Ozyesil Z, Deniz M, Dilmac B (2013). The prediction level of mindfulness and locus of control on subjective well-being. Int J Acad Res..

[59] Ariso JM, Reyero D (2014). Reconsidering the scenario of cyberbullying: promoting the internalization of the locus of control in adolescents through cognitive restructuring. Adolesc Psychiatry.

[60] Bloo J, Arntz A, Schouten E (2017). the borderline personality disorder checklist: psychometric evaluation and factorial structure in clinical and nonclinical samples. Roczniki Psychologiczne.

